# Hæmorrhoids: Causes and Symptoms

**Published:** 1896-02-01

**Authors:** A. G. Miller

**Affiliations:** Lecturer on Clinical Surgery, Senior Surgeon Edinburgh Royal Infirmary.


### MEDICAL PROGRESS AND HOSPITAL CLINICS.

[*The Editor will be glad to receive offers of co-operation and contributions from members of the profession. All letter should be addressed to* THE EDITOR, AT THE OFFICE, 428, STRAND, LONDON, W.C.]

### HÆMORRHOIDS—CAUSES AND SYMPTOMS.

By A. G. MILLER, M.D., F.R.C.S.E., Lecturer on Clinical Surgery, Senior Surgeon Edinburgh Royal Infirmary.

(*Continued from page* 281.)

But it may be urged that these are not the symptoms of the patients who present themselves for treatment at a hospital. That is true. I have been describing the early symptoms of a first attack, the beginning of a case of piles. Hospital patients present themselves for treatment only after they have suffered for a long time, and have become advanced, chronic, or aggravated cases. Their symptoms are, mainly, two, (1) something coming down, and (2) bleeding.

1. Something coming down at stool which has to be put up again is, of course, very characteristic of internal piles, of which I shall speak mainly now. If something comes down, there must be something to come down, and that is most likely to be a pile, or a mass of piles, for prolapse of the rectum and polypus are uncommon. The patient will tell us that the thing that comes down went back of its own accord at first, when defæcation was completed. After a while, however, the piles remained down and had to be returned by the hand. Then a time came when they would not return, but remained external to the anus. Internal piles, when they have reached this stage, are often called external piles, but they are not so, properly speaking. They are submucous, and are only, as it were, accidentally outside the anus—dislocated. When first protruded they are dark-coloured and glossy, being covered by normal mucous membrane, but after a time this becomes thickened, and loses its gloss, and becomes like skin. On examination, however, the pile can be made out as having its root or origin above the line of junction of the skin and mucous membrane.

2. The second symptom is bleeding. This bleeding has certain characteristics—it is venous, it occurs at the termination of stool, and it ceases when the piles are returned within the sphincter. Sometimes a considerable amount of blood is lost, and consequently, if bleeding occurs frequently, anæmia of a very intense character is produced, which I may appropriately refer to here. As this anæmia is due to direct loss of blood, it is general, affecting every part and organ in the body, and is easily cured by stopping the bleeding. Iron and tonics, of course, do no good while the bleeding piles are untreated. The symptoms of this anæmia are frequently misunderstood by the patient. He will say that his head is bad, he has giddiness and singing in the ears, his eyesight is bad, his heart is wrong, he has palpitations, his lungs (he says) are weak, he cannot make the least exertion without bringing on breathlessness, his liver is wrong, and he suffers from constipation. He has forgotten to care about his piles, or perhaps he looks upon them as a blessing—a kind of safety valve—because if they do not bleed for some days he becomes uncomfortable, and has a headache, but when the bleeding occurs he is much relieved. In this way some have nursed their disease until it has proved fatal. Whenever, therefore, there is general anæmia in an otherwise healthy person, suspect bleeding piles. But how do piles bleed? The explanation is simple. From continued irritation, the pile becomes not only inflamed, but ulcerated, and the ulceration, destroying the mucous membrane, opens into the vein forming the pile, which has, unfortunately, not been obliterated by thrombosis. Why the bleeding occurs only at defæcation, and ceases when the pile is returned, may be explained thus—the opening in the vein closes readily. If this were not so, the bleeding once started would be continuous. Before the act of defæcation the opening is closed, but when the pile is extruded and pressed upon, and rubbed by the fæces, the ulcer is burst open. Then bleeding occurs, and continues so long as the pile is "down." When the pile is returned it is at once compressed by the sphincter, the bleeding stops, and the rupture gets time to heal. Bleeding does not occur every time the patient goes to stool, which, of itself, proves that the tear in the vessel heals. Sometimes the loss of blood is slight (a teaspoonful), sometimes severe (an ounce or two); the amount depends mainly on the length of time that the piles are allowed to remain down.

3. Pain is, of course, a symptom of piles, but markedly only in the inflamed form, more especially the external variety. In the chronic old-standing internal piles, the pain is most conspicuous by its absence. If pain exist in these cases, it is most likely due to a complication, viz., fissure. This suggests the consideration of diagnosis.

*C.* The diagnosis of an external pile, for these troublesome and painful things are usually (fortunately) single, is not difficult. It is a painful lump, external to the anus and close to it, and when felt or seen is generally easily recognised. The diagnosis of internal piles, however, requires more careful consideration. They may be confounded with a variety of affections, especially by the patients themselves. (*a*) Polypus of the rectum differs from piles in having little or no bleeding, and no history of pain. Something comes down, but when seen this something, which is always single, is pinkish or white, has a long pedicle, and when ligatured and cut off gives rise to no further trouble. (*b*) Prolapse of the rectum is circular, and, when seen cannot be mistaken for anything but what it is—bowel, though its mucous membrane may be much changed by long exposure and irritation. (*c*) Fissure of the anus is a condition often confounded with, or mistaken for piles. It is a small painful ulcer at the margin of the anus. Its one very characteristic symptom is pain, coming on after stool, and continuing for an hour or many hours. It is frequently found at the base of a tag of skin that represents an old external pile. There may be bleeding from a fissure, but it is always slight, occurs at stool (not after), and the blood is sometimes on the fæces. Fissure is frequently associated with piles, because the cause is the same, viz., constipation. Sometimes also the same treatment cures both, viz., stretching the sphincter. (*d*) Fistula in ano, although it is not infrequently confounded with piles (by patients), has no symptom in common with piles—unless it be pain, which, however, is common to most diseases. The main symptom of fistula, after it is fully formed, is a discharge of pus, and sometimes of liquid fæces and flatus, from an opening or sinus near the anus. There is a history of an abscess which has burst or been opened some time before. (*e*) Cancer of the rectum has to be diagnosed from piles, because the unfortunate sufferers from the former disease not unfrequently are under the delusion that they have got only the latter affection, and because piles sometimes exist as a symptom of the more formidable malady. I need not enter at any length on the symptoms of malignant disease of the rectum; suffice it to say that those which should raise our suspicions are, alternating looseness and constipation, difficulty in defæcation associated with pain, or a sense of weight or obstruction, and the discharge of mucus or matter mixed with blood. (*f*) Piles may exist as a symptom of cirrhosis of the liver. In such a case attention should be directed to that organ, and not to the local affection.

I will close these remarks on diagnosis with the advice given by all writers on diseases of the rectum, viz., never to trust to symptoms, but always to make a thorough examination of the anus and rectum.

